# Unfolded Protein Response at the Crossroads: Integrating Endoplasmic Reticulum Stress with Cellular Stress Networks

**DOI:** 10.3390/ijms27041986

**Published:** 2026-02-19

**Authors:** Sebastian Gawlak-Socka, Edward Kowalczyk, Anna Wiktorowska-Owczarek

**Affiliations:** Department of Pharmacology and Toxicology, Medical University of Lodz, Zeligowskiego 7/9, 90-752 Lodz, Poland; sebastian.gawlak-socka@stud.umed.lodz.pl (S.G.-S.); edward.kowalczyk@umed.lodz.pl (E.K.)

**Keywords:** endoplasmic reticulum stress, unfolded protein response, oxidative stress, autophagy, inflammation, metabolic stress

## Abstract

The endoplasmic reticulum (ER) is a central hub of cellular proteostasis, coordinating protein folding, lipid metabolism, calcium signaling, and inter-organelle communication. Disruptions in ER function activate the unfolded protein response (UPR), an evolutionarily conserved signaling network mediated by PERK, IRE1α, and ATF6. Initially viewed primarily as a stress-mitigating mechanism, the UPR is now recognized as a central coordinator of diverse cellular stress-response pathways. This review focuses on mechanistic insights into UPR signaling, with particular emphasis on its crosstalk with oxidative stress regulation, mitochondrial function and mitochondria–ER contact sites, autophagy, inflammatory signaling, and metabolic sensing. The analysis integrates evidence from biochemical and structural studies, genetic and pharmacological perturbation models, and selected in vivo investigations from PubMed and Google Scholar between 2000 and 2025, focusing on mechanistic, experimental and translational studies addressing UPR signaling and ER stress. Together, these studies demonstrate how transient UPR activation promotes cellular adaptation through coordinated transcriptional, translational, and organelle-specific responses. We further discuss how sustained or unresolved ER stress alters UPR outputs, shifting signaling toward maladaptive outcomes such as mitochondrial dysfunction, dysregulated autophagy, oxidative imbalance, and apoptosis. By placing the UPR within a network of interconnected stress pathways, this work provides a framework for understanding how ER proteostasis is linked to cell fate decisions under stress.

## 1. Introduction

The endoplasmic reticulum (ER) is a central regulator of cellular homeostasis, managing protein folding, lipid biosynthesis, detoxification reactions, and intracellular trafficking [1]. Its expansive network of rough and smooth domains underlies both: the biosynthetic and quality control processes required to sustain proteostasis in eukaryotic cells. Because a substantial proportion of nascent polypeptides undergo maturation within the ER, this organelle is exquisitely sensitive to physiological and environmental perturbations that elevate the burden of newly synthesized proteins [2]. A wide array of stressors—including hypoxia, redox imbalance, inflammation, nutrient limitation, xenobiotic exposure, and heightened proliferative demand can exceed the ER’s folding capacity, leading to the accumulation of misfolded or incompletely processed proteins, a condition collectively referred to as ER stress [3].

To restore ER functionality, cells engage the unfolded protein response (UPR), an evolutionarily conserved signaling network governed by three principal ER-resident sensors: Inositol-Requiring Enzyme 1 (IRE1), Protein Kinase RNA-like Endoplasmic Reticulum Kinase (PERK), and Activating Transcription Factor 6 (ATF6) [4,5]. Under basal conditions, these sensors are maintained in an inactive state through association with the master chaperone GRP78/BiP. Accumulation of misfolded proteins sequesters glucose-regulated protein 78 (GRP78), thereby permitting activation of the UPR and the induction of adaptive transcriptional and translational programs that enhance the ER’s folding capacity, reduce global protein synthesis, and promote clearance of terminally misfolded proteins via ER-associated degradation [6]. Although the UPR is fundamentally cytoprotective, sustained or excessive ER stress can divert this response toward maladaptive outcomes, including apoptosis, inflammatory signaling, metabolic dysfunction, and disruption of organelle communication particularly at mitochondria-ER contact sites (MERCs) [7].

Accumulating evidence indicates that the UPR operates not as an isolated pathway but as a nodal integrator within a broader network of cellular stress-response systems. ER stress functionally crosses with redox regulation, mitochondrial quality-control pathways, autophagy, innate immune signaling, and multiple forms of regulated cell death, collectively shaping cell fate decisions under stress [7]. Perturbation or chronic dysregulation of these connected pathways contributes to the initiation and progression of diverse human diseases, including cancer, neurodegenerative disorders, cardiometabolic diseases, and inflammatory pathologies [8]. Environmental exposures such as cigarette smoke and its constituents, including nicotine, further modulate ER homeostasis by altering GRP78 levels and reshaping UPR signaling, underscoring the sensitivity of this system to extrinsic cues [9].

Given the centrality of UPR signaling cellular adaptation and disease pathogenesis, a refined understanding of how ER stress interfaces with other stress-response networks are essential for identifying new therapeutic targets. In this review, we focused on recent mechanistic insights into UPR biology with emerging concepts in ER-centered cross-organelle communication. We characterize the molecular logic, context-dependent consequences, and translational implications of UPR signaling, with the goal of defining how this pathway functions as a regulatory nexus in human health and disease.

## 2. Aim and Scope of the Review

Current research on ER stress and UPR signaling has substantially expanded, yet the field remains dominated by studies focusing on individual UPR branches or specific downstream mechanisms rather than integrated regulatory networks. While these reductionist approaches have provided invaluable mechanistic insights, they often fail to capture the integrated nature of ER stress responses as they operate within the broader cellular stress landscape. Consequently, a comprehensive framework that connects UPR signaling with organelle-specific processes and parallel stress-response systems is still lacking.

The aim of this review is to bridge this gap by providing an integrated overview of UPR signaling as a central regulatory network that coordinates cellular adaptation to stress. Rather than treating PERK, IRE1α, and ATF6 as independent pathways, we emphasize their functional interplay and contextual crosstalk with oxidative stress regulation, mitochondrial function and MERCs, autophagy, inflammatory signaling, and metabolic sensing. By synthesizing findings from biochemical, genetic, imaging, and translational studies, we highlight how these interconnected processes collectively shape cellular homeostasis and fate decisions. The novelty of this review lies in its systems-level perspective, which positions the UPR not merely as a response to protein misfolding, but as an integrative stress-adaptation platform linking ER proteostasis to broader cellular and organelle-specific signaling networks.

## 3. The Endoplasmic Reticulum: Structure, Function and Proteostasis Control

The ER is the largest membrane-bound organelle in eukaryotic cells and plays a central role in maintaining cellular homeostasis by coordinating protein folding, lipid biosynthesis, calcium storage, and intracellular trafficking [10]. Its extensive and dynamic membrane network supports both biosynthetic and quality-control functions, making the ER a key determinant of proteostasis. Because a substantial proportion of nascent polypeptides undergo folding and maturation within the ER lumen, this organelle is particularly sensitive to fluctuations in metabolic demand and environmental conditions [11].

A principal function of the ER is the synthesis, folding, and maturation of secretory and membrane proteins. Nearly one-third of the cellular proteome transits through the ER, necessitating a highly efficient proteostasis network composed of molecular chaperones, oxidoreductases, and folding enzymes [12,13]. Among these, the chaperone GRP78/BiP plays a central regulatory role by binding unfolded polypeptides, preventing premature export of misfolded proteins, and coordinating the activation of ER stress–responsive signaling pathways [6,12].

To preserve proteome integrity, the ER employs complementary quality-control mechanisms that eliminate terminally misfolded proteins. ER-associated degradation (ERAD) retro-translocates aberrant proteins into the cytosol for ubiquitin–proteasome–mediated degradation, while ER-phagy, a selective form of autophagy, removes aggregated proteins or damaged ER subdomains via lysosomal pathways [13]. Together, these systems enable the ER to adapt to fluctuating folding demands and maintain functional homeostasis.

Beyond proteostasis, the ER contributes to lipid metabolism, calcium signaling, and inter-organelle communication. Of particular relevance are MERCs, specialized domains that facilitate the exchange of calcium ions, lipids, and signaling metabolites between the ER and mitochondria [14,15]. These interfaces play a critical role in coordinating metabolic activity, redox balance, and cell survival decisions, and become especially important under conditions of ER stress.

Given its central role in handling a large fraction of the cellular proteome, the ER is highly sensitive to stressors such as hypoxia, oxidative imbalance, inflammation, nutrient limitation, and increased biosynthetic demand. When the burden of newly synthesized or misfolded proteins exceeds the folding capacity of the ER, proteostasis is disrupted and ER stress ensues, triggering activation of the UPR [16]. In this manner, the ER functions not only as a biosynthetic platform but also as a stress-sensing organelle that dynamically adjusts cellular physiology to preserve homeostasis.

## 4. Unfolded Protein Response Signaling Pathways: A Modern Overview

**Figure 1:**
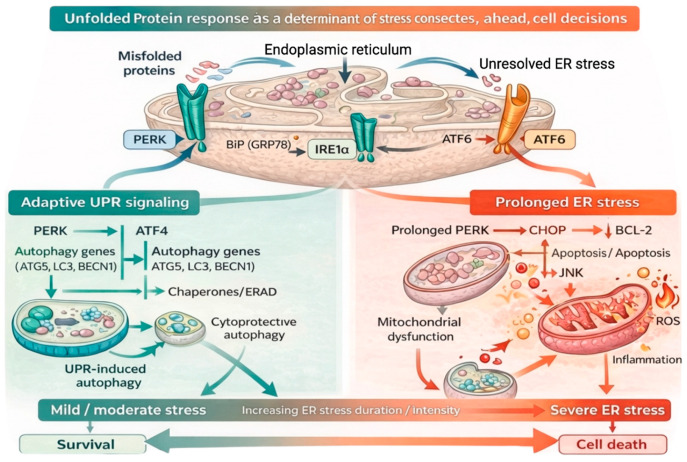
The unfolded protein response as a central regulator of adaptive and maladaptive stress responses [18]. Accumulation of misfolded or unfolded proteins within the ER triggers activation of the UPR through its three canonical branches: PERK, IRE1α, and ATF6. During early or moderate ER stress, coordinated activation of these pathways promotes adaptive responses aimed at restoring proteostasis. These include attenuation of global protein synthesis to reduce ER load, induction of chaperone expression, expansion of ER capacity, and activation to remove misfiled proteins and damaged organelles, thereby supporting cell survival. In contrast, prolonged or unresolved ER stress leads to a qualitative shift in UPR signaling. Sustained PERK–ATF4–CHOP signaling and prolonged IRE1α–JNK activation promote mitochondrial dysfunction, oxidative imbalance, and apoptotic pathways, whereas transient IRE1α–JNK signaling can contribute to adaptive autophagy and short-term cell survival. Autophagy, initially cytoprotective, may transition to a pro-death mechanism under chronic stress conditions. Through these context-dependent outputs, the UPR integrates ER proteostasis with mitochondrial function, autophagy, and cell fate determination.

The UPR pathway consists of three major signaling cascades initiated by the ER transmembrane stress sensors IRE1, ATF6, and PERK. These sensors contain luminal domains that detect the accumulation of unfolded polypeptides, as well as cytosolic regions that transmit signals through transcriptional and translational machinery to protect the cell from ER stress under physiological conditions [17]. Figure 1 illustrates the dual nature of UPR signaling, emphasizing that the same molecular pathways can mediate either adaptive or maladaptive outcomes depending on stress intensity and duration. The schematic highlights how early UPR activation prioritizes restoration of ER function and cellular survival, whereas persistent signaling progressively engages mitochondrial dysfunction and apoptotic programs. This dynamic transition underscores the role of the UPR not as a binary on–off response, but as a flexible regulatory system that continuously integrates ER stress signals with downstream organelle-specific and cell fate pathways.

### 4.1. PERK

During mild to moderate ER stress, adaptive responses are initiated within minutes, and one of the earliest is a transient reduction in global translation [7]. This decrease in protein synthesis serves a straightforward purpose: it limits the flow of nascent polypeptides into the stressed ER, buying the cell time to restore folding capacity. The PERK–eIF2α axis is central to this response and is activated when accumulated unfolded proteins promote PERK oligomerization and transautophosphorylation, which then leads to phosphorylation of the eukaryotic initiation factor 2α (eIF2α) α-subunit [3,8].

Although phosphorylated eIF2α suppresses most cap-dependent translation, it simultaneously enhances the synthesis of selected transcripts containing upstream open reading frames. Activating transcription factor 4 (ATF4) is the most prominent among them and functions as a transcriptional hub coordinating amino acid metabolism, oxidative stress defense, and broader stress adaptation programs [19]. Through these outputs, ATF4 broadens the range of processes that PERK can influence, temporarily redirecting metabolic resources toward damage control.

An important aspect of this branch of the UPR is its built-in feedback architecture. ATF4 induces growth arrest and DNA damage-inducible protein 34 (GADD34), the stress-regulated cofactor of protein phosphatase 1 (PP1), which dephosphorylates eIF2α once ER conditions begin to improve [20]. Together with the constitutive PP1 cofactor CReP, GADD34 ensures that the translational block remains reversible and does not persist longer than necessary, preventing harmful consequences of prolonged protein-synthesis inhibition [21].

With more sustained or severe stress, however, the PERK pathway shifts toward pro-apoptotic signaling. ATF4 drives the expression of CHOP, which lowers the threshold for mitochondrial apoptosis by repressing anti-apoptotic B-cell lymphoma 2 (BCL-2) family proteins, promoting oxidative stress, and sensitizing the mitochondria to membrane permeabilization [7]. Beyond translation control, PERK also affects mitochondrial function at MERCs, influencing Ca^2+^ transfer, bioenergetics, and redox balance. These connections help explain how this pathway toggles between adaptation and commitment to cell death [22].

A study by Kopp et al. [19] provided additional information on how PERK couples to the ER chaperone network. The authors showed that PERK activation is closely linked to the ATPase cycle of BiP/GRP78, the major luminal sensor of unfolded proteins. In a reconstituted human chaperone system, ERdj3, a J-domain-containing co-chaperone that stimulates BiP ATP hydrolysis and Sil1, a nucleotide exchange factor that promotes ADP-ATP exchange on BiP, stimulated BiP’s basal ATPase activity, as expected for Hsp70-class chaperones. Interestingly, although the luminal domains of PERK or IRE1 did not alter BiP’s intrinsic ATPase rate on their own, their addition reduced the co-chaperone-stimulated enhancement, essentially shifting the activity back toward baseline.

These observations suggest that UPR sensor luminal domains do not directly modulate BiP catalysis but can influence how co-chaperones engage BiP, likely by competing for binding or altering the conformation available for client interactions. This provides an additional layer of control linking luminal stress detection to the earliest steps in PERK activation and reinforces the idea that BiP acts both as a folding chaperone and as a regulatory gatekeeper of the UPR [23].

### 4.2. IRE1α

Regulation of the UPR is also mediated by IRE1α, which activates its endoribonuclease function upon oligomerization and autophosphorylation [24]. This activation triggers the unconventional splicing of X-box binding protein 1 (XBP1) mRNA, producing the active transcription factor XBP1s, which in turn upregulates genes involved in ER protein translocation, folding, secretion, and the degradation of misfolded proteins. This splicing event occurs at the micron-scale level, wherein a short intron is excised from XBP1 mRNA, thereby shifting the translational reading frame to generate the functional transcription factor [25,26].

Notably, the RNase activity of IRE1α extends beyond canonical XBP1 mRNA splicing. Under conditions of sustained ER stress, IRE1α engages in a broader endonucleolytic program that includes the selective cleavage of microRNA precursors. This process, demonstrated for several miRNAs such as miR-17, miR-34a, and miR-96, leads to their degradation and the subsequent derepression of target mRNAs. One key consequence of this mechanism is the enhanced translation of caspase-2, which functions as an initiator of caspase under conditions of unresolved ER stress, thereby linking prolonged IRE1α signaling to the activation of apoptotic pathways [27]. This expanded substrate repertoire of IRE1α highlights its dual capacity to promote adaptive remodeling during early stress while simultaneously priming apoptotic programs when ER dysfunction becomes irreversible.

In addition to its role in XBP1 mRNA splicing, IRE1α engages in a second RNase-driven pathway known as regulated IRE1-dependent decay (RIDD). Through this mechanism, IRE1α selectively cleaves a subset of ER-associated mRNAs, thereby decreasing the load of nascent polypeptides entering the ER and helping to restore folding equilibrium. Although the quantitative contribution of RIDD to global proteostasis remains incompletely defined, accumulating evidence shows that its effects are highly context-dependent, varying with cell type, stress intensity, and metabolic state [28,29].

Substrates of RIDD often share a conserved sequence motif (CUGCAG) embedded within a defined stem-loop structure, which is recognized by the IRE1α RNase domain. Beyond mRNA decay, RIDD can influence the abundance of other RNA species, including select pre-microRNAs, thereby shaping post-transcriptional gene regulation during ER stress [30].

Recent structural and biochemical studies have demonstrated that IRE1α physically associates with components of the translational and translocation apparatus, including the signal recognition particle, ribosomal RNA, and tRNAs [31]. These interactions raise the possibility that RIDD may be spatially coordinated with sites of active translation or co-translational translocation, although the physiological significance of these IRE1α, RNA complexes remains to be fully elucidated. Moreover, IRE1α forms complexes with various adaptor proteins that facilitate its integration into broader stress-responsive networks. Through these interactions, IRE1α can modulate autophagy, engage MAP kinase pathways (MAPK), and influence additional signaling modules that determine whether the cell adopts adaptive or pro-death outcomes in response to persistent ER stress [32].

### 4.3. ATF6

The activation mechanism of ATF6, the third branch of the UPR, differs fundamentally from that of PERK and IRE1α. Instead of initiating signaling through catalytic activity in the ER membrane, ATF6 operates as a transport-dependent sensor. Upon accumulation of unfolded proteins, ATF6 is released from its association with ER chaperones and traffics to the Golgi apparatus, where it undergoes sequential cleavage by Site-1 and Site-2 proteases (S1P and S2P) [33]. This regulated intramembrane proteolysis generates the soluble cytosolic fragment ATF6p50, which translocates to the nucleus to modulate transcription. Once activated, ATF6p50 drives the expression of a broad set of genes that enhance ER proteostasis capacity. Together with XBP1s, it induces the transcription of ER-resident chaperones, components of the protein translocation machinery, and enzymes responsible for proper folding, maturation, and secretion of nascent polypeptides [34].

Beyond their classical roles in proteostasis, ATF6 and XBP1s also contribute to ER and Golgi biogenesis, increasing the structural and functional capacity of the secretory pathway. This remodeling is particularly evident under conditions of persistent or moderate-to-severe ER stress, when cells require enhanced secretory output and expanded folding machinery to maintain viability [7,35].

These three branches of the unfolded protein response differ not only in their modes of activation and core molecular outputs, but also in how they intersect with broader cellular stress-response networks. As summarized in Table 1, PERK, IRE1α, and ATF6 engage distinct yet overlapping signaling modules that coordinate translational control, RNA metabolism, proteostasis, autophagy, inflammation, and mitochondrial function. Importantly, the functional consequences of these signaling programs are highly context dependent. As outlined in Table 2, each UPR branch can promote either adaptive or maladaptive outcomes depending on stress intensity and duration, highlighting how the same signaling axes may support cell survival under transient stress or drive inflammation, organelle dysfunction, and cell death when ER stress remains unresolved.

## 5. Crosstalk Between the UPR and Other Cellular Stress Pathways

The UPR functions as a central regulatory hub that coordinates multiple cellular protective mechanisms. It detects disturbances in ER proteostasis and integrates this information to orchestrate adaptive responses across several stress-response networks. Through its broad signaling influence, the UPR regulates key axes of cellular homeostasis, including oxidative and redox signaling, mitochondrial function, and MERC, autophagy, inflammatory signaling and metabolic sensing. Together, these interconnected pathways significantly shape cellular fate and have profound implications for the pathogenesis of numerous diseases.

The major point of integration between UPR signaling and other cellular stress-response pathways, along with their disease-related consequences, are summarized in Table 3.

### 5.1. Integration of UPR with Oxidative Stress Responses

**Figure 2:**
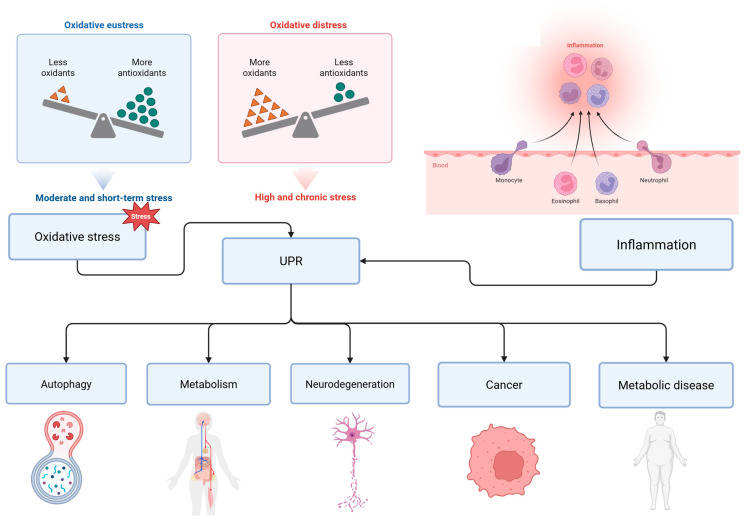
The UPR as an integrative hub linking oxidative stress, inflammation, and disease outcomes [51]. This figure depicts the bidirectional relationship between oxidative stress and unfolded protein response signaling. Moderate and transient stress promote oxidative eustress, characterized by a reactive oxygen species (ROS) production balanced by antioxidant defenses. Under these conditions, UPR activation supports adaptive responses, including cytoprotective autophagy, metabolic homeostasis and restoration of redox balance. In contrast, chronic or high-intensity stress shifts this balance toward oxidative distress, marked by excessive ROS accumulation and insufficient antioxidant defenses. Persistent oxidative stress amplifies the UPR, which in turn modulates inflammatory pathways, metabolic dysregulation and cell death mechanisms. Sustained or dysregulated UPR activity contributes to pathological outcomes such as neurodegeneration, cancer, and metabolic disease. Reciprocal interactions between UPR signaling and inflammation further amplify stress responses, shaping cell fate decisions, and disease progression.

ER protein folding is an intrinsically redox-active process that generates reactive oxygen species (ROS). Consequently, ER stress and oxidative stress are tightly related. Figure 2 places the UPR within a broader network of redox and inflammatory signaling, highlighting its function as a central stress-integration hub rather than an isolated ER-specific pathway. By distinguishing between oxidative eustress and oxidative distress, the figure clarifies how transient UPR activation supports homeostatic adaptation, whereas chronic stress drives feed-forward loops involving ROS accumulation and inflammatory signaling. This conceptual framework helps explain why prolonged ER stress is strongly associated with chronic inflammatory states and degenerative diseases. Activation of the PERK branch promotes antioxidant defenses in part via direct phosphorylation and activation of nuclear factor erythroid 2-related factor 2 (NRF2), which increases transcription of genes involved in glutathione biosynthesis and redox buffering, thereby mitigating ROS accumulation during acute ER stress [36]. The other way—prolonged IRE1α signaling, via c-Jun N-terminal Kinase (JNK) and other effectors, can augment oxidative damage and tip the balance toward cell death if antioxidant responses are insufficient [38]. These reciprocal interactions place the UPR as a key regulator of cellular redox homeostasis and a potential therapeutic node in redox-driven pathologies.

### 5.2. Integration of UPR with Mitochondrial Function and MERC

Physical and functional contacts between the ER and mitochondria, mitochondria–ER contact sites (MERCs), provide platforms for Ca^2+^ transfer, lipid exchange, and localized signaling that coordinate bioenergetics and survival decisions. PERK influences MERC integrity and modulates Ca^2+^ flux into mitochondria, thereby affecting ATP production and susceptibility to mitochondrial outer membrane permeabilization under stress [38].

Importantly, mitochondrial proteins are synthesized exclusively on free cytosolic ribosomes and delivered directly to mitochondria post-translationally. Emerging evidence indicates that the endoplasmic reticulum actively participates in this process by acting as a transient staging platform for newly synthesized mitochondrial precursor proteins. The ER-SURF (ER Surface–mediated protein targeting) pathway demonstrates that a subset of mitochondrial-destined proteins initially associates with the ER membrane, where ER-resident chaperones facilitate their stabilization and subsequent transfer to mitochondria. This mechanism becomes particularly relevant under stress conditions when global cytosolic translation and protein folding capacity are compromised. Within this framework, MERCs function as privileged zones that spatially couple protein synthesis, quality control, and mitochondrial import. UPR activation may therefore coordinate cytosolic stress responses with mitochondrial proteostasis by redirecting nascent polypeptides and associated mRNAs toward ER membranes and MERCs, ensuring efficient mitochondrial protein supply while limiting proteotoxic stress in the cytosol. These findings position the ER not merely as a passive signaling partner of mitochondria, but as an active participant in mitochondrial protein biogenesis and stress adaptation [39].

A study conducted by Pihán et al. [40] demonstrated that ER stress induces the redistribution of specific mitochondrial mRNAs, including transcripts encoding components of the respiratory chain and mitochondrial chaperones, toward MERCs, where active ribosomes remain engaged in translation. This spatial reprogramming ensures that mitochondria continue to receive essential proteins required for maintaining oxidative phosphorylation, membrane potential, and ROS detoxification during stress. The authors propose that this mechanism represents a highly conserved adaptive response, allowing cells to preserve mitochondrial fitness even when global translation is suppressed. Their findings also imply that MERCs may act as translational sanctuaries, integrating local signaling, RNA trafficking, and protein biogenesis into a unified stress–response platform. Moreover, chronic IRE1α/RIDD or sustained PERK activity can engage mitochondrial apoptotic pathways, linking prolonged ER dysfunction with mitochondrial-driven cell death.

### 5.3. Integration of UPR with Autophagy

Autophagy operates in tight coordination with the UPR to restore proteostasis during prolonged ER stress. All three branches of the UPR modulate autophagy through distinct transcriptional and post-translational mechanisms, and together they determine whether autophagy functions as an adaptive, cytoprotective process or shifts toward a pro-death pathway.

#### 5.3.1. PERK-eIF2α-ATF4 Axis as a Master Transcriptional Inducer of Autophagy

Activation of PERK triggers phosphorylation of eIF2α, which—besides attenuating global translation—preferentially enhances translation of ATF4, a transcription factor central for autophagy induction. ATF4 directly increases transcription of several autophagy-related (ATG) genes, including ATG5, ATG7, ATG12, LC3B (MAP1LC3B), and BECN1, thereby supporting formation of autophagosome machinery [40,41]. ATF4 also induces CHOP, which cooperates with ATF4 on specific promoters to amplify expression of autophagy genes such as TRIB3, GABARAPL1, and DDIT3, particularly during prolonged or severe ER stress [42]. Through TRIB3-mediated inhibition of Akt, the PERK–ATF4–CHOP pathway can additionally suppress mTORC1—a major negative regulator of autophagy, thus promoting autophagic flux [43]. Another mechanistic layer involves ATF4-dependent stimulation of amino acid metabolism and redox-protective pathways, which support sustained autophagic activity by maintaining intracellular nutrient and redox balance under proteotoxic stress [44].

#### 5.3.2. IRE1α-TRAF2-JNK Pathway and the Regulation of Autophagy Initiation

The second UPR branch contributes to autophagy predominantly through IRE1α-mediated recruitment of tumor necrosis factor receptor-associated factor 2 (TRAF2), which activates JNK. JNK phosphorylates BCL-2, releasing Beclin-1, a crucial autophagy initiator, from inhibitory BCL-2 complexes [45]. This permits assembly of the Beclin-1-Vps34 PI3K complex, thereby initiating autophagosome nucleation. RE1α-dependent RIDD may further influence autophagy by selectively degrading transcripts encoding ER-targeted proteins, thereby reducing the proteotoxic burden on the ER and facilitating adaptive autophagic responses [49]. In specific contexts, RIDD also degrades mRNAs encoding negative regulators of autophagy, thereby favoring autophagic activation. Chronic or hyperactivated IRE1α signaling, however, may shift the balance toward cell death, partly by JNK-driven mitochondrial outer membrane permeabilization or by RIDD-mediated decay of transcripts crucial for survival [29].

### 5.4. Integration of UPR with Inflammation

The UPR modulates inflammatory signaling at multiple regulatory levels, acting both upstream and downstream of classical immune pathway. Among the three UPR branches, IRE1α plays a central role in linking ER stress to inflammation through its interaction with tumor necrosis factor receptor–associated factor 2 (TRAF2), leading to activation of NF-κB and AP-1 transcriptional programs. These pathways promote the expression of pro-inflammatory cytokines, chemokines, and stress-response genes, thereby coupling ER proteostasis to innate immune activation [5,49].

PERK signaling further shapes inflammatory responses by modulating translational output and redox homeostasis. PERK-mediated phosphorylation of eIF2α selectively suppresses global protein synthesis while permitting translation of stress-responsive transcription factors, including ATF4, which can influence cytokine production, oxidative stress responses, and inflammasome activation. ATF6-dependent transcriptional remodeling also contributes to inflammatory regulation by altering ER folding capacity and secretory pathways, indirectly affecting the synthesis and release of immune mediators [52].

In immune cells, UPR signaling has context-dependent functions that extend beyond stress mitigation. IRE1α and spliced XBP1 are critical for macrophage polarization, dendritic cell maturation, antigen presentation, and metabolic reprogramming during immune activation. Sustained or dysregulated UPR–inflammatory crosstalk contributes to pathological conditions characterized by chronic inflammation, including metabolic disorders, neuroinflammatory diseases, and tumor-promoting inflammatory microenvironments. Together, these findings position the UPR as a key regulator of inflammatory tone, bridging cellular stress responses with innate and adaptive immunity [52,53].

### 5.5. Integration of UPR with Neurodegenerative Diseases

Neurodegenerative diseases represent a pathological context in which persistent ER stress and failure of UPR resolution play a particularly prominent role. Chronic activation of the PERK–eIF2α axis is a recurring feature across multiple neurodegenerative conditions, leading to sustained translational repression and progressive impairment of neuronal proteostasis. In postmitotic neurons, prolonged suppression of protein synthesis compromises synaptic maintenance and plasticity, thereby contributing to early synaptic dysfunction [7,54].

In parallel, defective resolution of ER stress exacerbates mitochondrial dysfunction and oxidative imbalance, partly through disrupted ER–mitochondria communication and altered calcium handling. These alterations amplify neuroinflammatory signaling and further destabilize cellular homeostasis. Over time, the convergence of translational shutdown, impaired protein quality control, mitochondrial failure, and chronic inflammation culminates in neuronal loss, manifesting clinically as cognitive decline and motor dysfunction characteristic of neurodegenerative disease progression. Thus, maladaptive UPR signaling provides a unifying mechanistic framework linking ER stress to synaptic failure and neurodegeneration, as outlined in Table 3 [55].

### 5.6. Integration of UPR with Metabolic Sensors

Protein folding and secretion impose substantial energetic and biosynthetic demands, necessitating tight coordination between ER stress signaling and cellular metabolic status. The unfolded protein response interfaces with major metabolic sensors to align proteostatic capacity with nutrient availability and energy balance. PERK-mediated translational attenuation represents an immediate adaptive mechanism that reduces ATP consumption and conserves amino acids under conditions of ER stress. Concurrently, ATF4-driven transcriptional programs enhance amino acid import, biosynthesis, and redox buffering, thereby supporting stress adaptation [47].

The IRE1α–XBP1s axis plays a prominent role in regulating lipid metabolism and membrane biogenesis, particularly in highly secretory cells such as hepatocytes, plasma cells, and endocrine tissues. XBP1s promote phospholipid synthesis, ER membrane expansion, and lipid remodeling, ensuring sufficient ER capacity to accommodate increased secretory load. These processes are tightly integrated with nutrient-sensing pathways, including mechanistic target of rapamycin and AMP-activated protein kinase (AMPK), which coordinate anabolic and catabolic responses depending on cellular energy status [48,50].

Crosstalk between UPR signaling and metabolic regulators is especially relevant in metabolic disease contexts, where chronic ER stress disrupts insulin signaling, lipid homeostasis, and mitochondrial function. Persistent activation of UPR pathways can exacerbate metabolic imbalance, contributing to insulin resistance, hepatic steatosis, and systemic metabolic dysfunction. Thus, integration of the UPR with metabolic sensors represents a critical axis through which cells adapt—or maladapt—to prolonged stress, linking ER homeostasis to whole-organism metabolic health [47,48].

## 6. UPR as a Determinant of Cell Fate: Survival vs. Death

The UPR governs the decision between adaptive survival and programmed cell death in a manner dependent on the intensity, duration, and cellular context of ER stress [7]. This fate choice is shaped by multiple factors, including the interplay among the three canonical UPR branches—IRE1α, ATF6 and PERK, as well as organelle-specific processes such as autophagy and mitochondrial integrity, and broader signaling networks encompassing oxidative stress, inflammatory pathways, and metabolic sensors [32,50].

These adaptive outputs intersect with and reshape multiple cellular stress circuits. MERCs facilitate calcium transfer, lipid exchange, and localized translation, serving as regulatory nodes that either support mitochondrial fitness or amplify mitochondrial apoptotic signaling when stress is prolonged [39]. Both PERK and IRE1α are enriched at MERCs, where they influence mitochondrial dynamics, ROS production, and apoptotic sensitivity. Under persistent stress, PERK–ATF4–CHOP signaling increases oxidative burden and suppresses BCL-2 family survival factors, tipping the balance toward mitochondrial outer membrane permeabilization [54].

Autophagy also plays a dual role. Initially, UPR-dependent activation of autophagy supports cellular adaptation by clearing misfolded proteins and damaged organelles, reducing proteotoxic load, and restoring metabolic balance. ATF4 induces autophagy genes, IRE1α recruits TRAF2 to activate JNK-mediated autophagic processes, and ATF6 contributes indirectly by enhancing protein folding capacity and ER maintenance [33]. However, when ER stress cannot be resolved, autophagy transitions from a protective to a pro-death mechanism, amplifying cell elimination rather than preventing it.

A similar principle governs oxidative stress and inflammatory responses. Adaptive PERK–NRF2 signaling boosts antioxidant defenses, while prolonged activation yields excessive ROS that sensitize mitochondria to apoptosis. IRE1α complexes with TRAF2 and NOD1/2, triggering JNK and NF-κB pathways that initially support survival yet may drive chronic inflammation and tissue damage when hyperactivated, as observed in metabolic and neurodegenerative diseases [47,55]. Thus, the commitment to either survival or death arises from the temporal and quantitative balance of UPR signaling. Transient, oscillatory, or low-level activation promotes repair, ER expansion, metabolic rewiring, and controlled autophagy, enabling restoration of proteostasis. In contrast, chronic or high-intensity activation converts the same signaling modules into drivers of apoptosis—via CHOP induction, sustained IRE1α RNase activity, JNK activation, mitochondrial dysfunction, and inflammatory escalation [56].

Ultimately, the UPR serves as a decision-making platform that integrates ER proteostasis cues with broader stress networks to determine cellular fate. Its ability to coordinate cross-organelle communication, regulate metabolic and inflammatory states, and dynamically shift between adaptive and apoptotic programs explains its central role in the pathogenesis of diseases such as diabetes, neurodegeneration, cancer, and cardiovascular disorders [57].

## 7. Conclusions

The UPR is a central mechanism by which cells preserve homeostasis in the face of ER stress. Through the coordinated action of its three signaling branches: PERK, IRE1α, and ATF6, the UPR integrates translational control, transcriptional remodeling, and post-transcriptional regulation to restore proteostasis and adapt cellular metabolism to stress. While these pathways initially promote survival by limiting protein load, enhancing folding capacity, and activating quality-control systems, their sustained activation can shift signaling toward inflammation, mitochondrial dysfunction, and apoptosis.

Importantly, UPR signaling does not operate in isolation. Its close functional links with mitochondria, autophagy, redox regulation, inflammatory pathways, and metabolic sensors position the UPR as a broader stress-integration hub rather than a linear response to protein misfolding. This interconnectedness explains how unresolved ER stress contributes to the progression of diverse pathologies, including metabolic disease, neurodegeneration, and cancer.

A deeper understanding of how UPR signaling balances adaptive and maladaptive outcomes, particularly through its interactions with organelle-specific processes, will be essential for identifying therapeutic strategies aimed at restoring proteostasis and improving cellular resilience under chronic stress conditions.

Understanding how ER stress responses can be precisely modulated represents an important objective for future research, as it may enable the development of approaches that favor cellular adaptation and survival under stress, or alternatively exploit UPR signaling to eliminate cells that rely on chronic ER stress for their persistence, such as cancer cells.

## Figures and Tables

**Table 1:** Activation mechanisms, core effectors, and stress-pathway crosstalk of the three branches of the UPR.

UPR Branch	Activation Mechanism/Activation Effect	Major Cellular Outputs	Crosstalk with Other Stress Pathways
PERK [3,7,8,19,20,21,22,23]	BiP/GRP78 dissociation → PERK oligomerization and autophosphorylation → eIF2α phosphorylation	Transient attenuation of global translation; selective ATF4 translation; regulation of amino acid metabolism and redox hemostasis; stress-adaptive transcription	Redox: NRF2 activation; Autophagy induction: ATF4/CHOP → ATG genes, TRIB3–Akt–mTORC1 axis; regulation of Ca^2+^ flux and mitochondrial bioenergetics at MERCs; pro-apoptotic signaling via CHOP-mediated repression of BCL-2 family proteins
IRE1α [24,25,26,27,28,29,30,31,32]	GRP78/BiP release and unfolded protein binding → oligomerization/autophosphorylation → RNase activation	XBP1 mRNA splicing and induction of ER folding, secretion and ERAD genes; selective mRNA and miRNA decay via RIDD	Autophagy: TRAF2–JNK → BCL-2 phosphorylation → Beclin-1 release; Inflammation: NF-κB/AP-1 signaling; RNA metabolism: selective mRNA/miRNA decay; MERCs: mitochondrial stress integration
ATF6 [33,34,35]	GRP78/BiP release → ER-to-Golgi trafficking → S1P/S2P cleavage → nuclear translocation of ATF6p50	Upregulation of ER chaperones and quality-control machinery; expansion of ER folding and secretory capacity; ER and Golgi biogenesis	Proteostasis: reinforcement of ER quality control; Inflammation: tuning of secretory and cytokine programs; Autophagy: indirect relief of proteotoxic stress

The UPR is mediated by three ER-resident stress sensors: PERK, IRE1α, and ATF6, that are activated upon dissociation from the luminal chaperone GRP78/BiP in response to unfolded protein accumulation. Although all three branches cooperate to restore ER proteostasis, they differ in their activation mechanisms, downstream molecular effectors, and modes of integration with broader cellular stress-response pathways. PERK primarily regulates translational control and redox homeostasis, IRE1α coordinates transcriptional and posttranscriptional remodeling of the secretory pathway and stress signaling, while ATF6 expands ER folding and secretory capacity through transcriptional reprogramming. Sustained activation of these pathways promotes extensive crosstalk with autophagy, inflammatory signaling, mitochondrial function, and regulated cell death pathways, thereby shaping adaptive versus maladaptive cellular outcomes under ER stress.

**Table 2:** Adaptive and maladaptive outcomes mediated by individual UPR branches [19,20,21,22,23,24,25,26,27,28,29,30,31,32,33,34,35].

UPR Branch	Adaptive Response	Maladaptive Response
PERK	Transient attenuation, antioxidant response (ATF4), metabolic adaptation	CHOP induction, apoptosis, oxidative imbalance
IRE1α	XBP1s-mediated ER expansion, lipid metabolism, ERAD	JNK activation, inflammation, RIDD-mediated mRNA decay
ATF6	Chaperone induction, ERAD enhancement, ER capacity expansion	Chronic stress sensitization, dysregulated proteostasis

Adaptive and maladaptive signaling outputs downstream of the three principal unfolded protein response branches: PERK, IRE1α, and ATF6. Transient or moderate activation of these pathways promotes cytoprotective responses, including translational attenuation, enhancement of protein folding capacity, activation of quality-control mechanisms, metabolic adaptation, and pro-survival autophagy. In contrast, sustained or unresolved ER stress shifts UPR signaling toward maladaptive outcomes, such as chronic inflammation, oxidative imbalance, mitochondrial dysfunction, impaired autophagic flux, and apoptosis. This comparison highlights the context-dependent nature of UPR signaling and underscores its dual role in cellular adaptation and disease pathogenesis.

**Table 3:** Integration of UPR signaling with cellular stress pathways and disease outcomes.

Cellular Stress Pathway/Disease Context	Dominant UPR Features	Key Pathogenic Mechanisms	Representative Cellular or Tissue Consequences
Oxidative stress [36,37]	PERK–eIF2α–ATF4–NRF2 axis activation; sustained IRE1α–JNK signaling under chronic stress	Impaired redox buffering; excessive ROS production; shift from adaptive antioxidant signaling to oxidative damage	Mitochondrial dysfunction; increased susceptibility to apoptosis; redox-driven cellular injury
Mitochondrial function and MERCs [38,39]	PERK and IRE1α enrichment at MERCs; stress-induced redistribution of mitochondrial mRNAs to MERCs	Dysregulated Ca^2+^ transfer; altered bioenergetics; engagement of mitochondrial apoptotic pathways under chronic stress	Reduced ATP production; mitochondrial outer membrane permeabilization; stress-induced cell death
Autophagy [39,40,41,42,43,44,45]	PERK–eIF2α–ATF4–CHOP induction of autophagy genes; IRE1α–TRAF2–JNK signaling; indirect ATF6 contribution	Excessive or defective autophagic flux; imbalance between cytoprotective and pro-death autophagy	Clearance of misfolded proteins during adaptive stress or promotion of cell death under unresolved ER stress
Inflammatory signaling [5,46]	IRE1α–TRAF2–NF-κB/AP-1 activation; PERK- and ATF6-dependent modulation of cytokine production; XBP1s in immune cells	Enhanced cytokine production; macrophage and dendritic cell reprogramming; chronic inflammation	Persistent inflammation; tissue damage; immune dysregulation
Metabolic stress and nutrient sensing [47]	PERK-mediated translational attenuation; ATF4-driven amino acid metabolism; XBP1s regulation of lipid metabolism	Energetic imbalance; dysregulated anabolic and catabolic signaling; altered metabolic homeostasis	Metabolic dysfunction; contribution to metabolic disease pathogenesis
Neurodegenerative disease [7,47,48]	Persistent ER stress; prolonged PERK–eIF2α signaling; impaired UPR resolution	Sustained translational repression; defective proteostasis; mitochondrial dysfunction; neuroinflammation	Synaptic failure; neuronal loss; cognitive and motor decline
Chronic inflammatory and metabolic disease [46,49,50]	IRE1α dominance; XBP1s-driven immune and metabolic remodeling; maladaptive UPR activation	Chronic inflammation; metabolic rewiring; failure to resolve ER stress	Tissue damage; disease progression; loss of homeostatic control

The UPR functions as a central integrator of multiple cellular stress-response pathways. Through coordinated activation of PERK, IRE1α, and ATF6 signaling branches, the UPR interfaces with oxidative stress regulation, mitochondrial function at MERCs, autophagy, inflammatory signaling, and metabolic sensing. While transient UPR activation promotes adaptive remodeling and cellular survival, chronic or unresolved ER stress shifts these same pathways toward maladaptive outcomes, including mitochondrial dysfunction, sustained inflammation, defective proteostasis, and programmed cell death. Dysregulation of UPR crosstalk with these stress networks contributes to the pathogenesis of neurodegenerative, metabolic, and inflammatory diseases.

## Data Availability

No new data were created or analyzed in this study. Data sharing is not applicable to this article.

## Abbreviations

The following abbreviations are used in this manuscript:

AKT	Protein kinase B
AMPK	AMP-activated protein kinase
AP-1	Activator protein 1
ATF4	Activating transcription factor 4
ATF6	Activating transcription factor 6
ATF6(N)	N-terminal active fragment of ATF6
ER	Endoplasmic Reticulum
UPR	Unfolded Protein Response
ATG	Autophagy-related genes
BECN1	Beclin-1
BiP	Binding immunoglobulin protein
BCL-2	B-cell lymphoma 2
CHOP	C/EBP homologous protein
CReP	Constitutive repressor of eIF2α phosphorylation
DDIT3	DNA damage-inducible transcript 3 (gene encoding CHOP)
eIF2α	Eukaryotic initiation factor 2 alpha
ERAD	Endoplasmic reticulum–associated degradation
ERdj3	Endoplasmic reticulum DnaJ homolog 3
ER-phagy	Selective autophagy of the endoplasmic reticulum
GADD34	Growth arrest and DNA damage-inducible protein 34
GABARAPL1	GABA type A receptor-associated protein-like 1
GRP78	Glucose-regulated protein 78
Hsp70	Heat shock protein 70
IRE1α	Inositol-requiring enzyme 1 alpha
JNK	c-Jun N-terminal kinase
LC3B	Microtubule-associated protein 1 light chain 3 beta
MAPK	Mitogen-activated protein kinase
MERCs	Mitochondria–endoplasmic reticulum contact sites
mTOR	Mechanistic target of rapamycin
mTORC1	Mechanistic target of rapamycin complex 1
NF-κB	Nuclear factor kappa B
NOD1/2	Nucleotide-binding oligomerization domain-containing protein ½
NRF2	Nuclear factor erythroid 2–related factor 2
PERK	PKR-like endoplasmic reticulum kinase
PI3K	Phosphoinositide 3-kinase
PP1	Protein phosphatase 1
RER	Rough endoplasmic reticulum
RIDD	Regulated IRE1-dependent decay
ROS	Reactive oxygen species
SER	Smooth endoplasmic reticulum
S1P	Site-1 protease
S2P	Site-2 protease
TRAF2	Tumor necrosis factor receptor–associated factor 2
TRIB3	Tribbles pseudokinase 3
Vps34	Class III phosphatidylinositol 3-kinase
XBP1	X-box binding protein 1
XBP1s	Spliced, active form of X-box binding protein 1
